# Identification of autophagy-related genes ATG18 subfamily genes in potato (*Solanum tuberosum* L.) and the role of *StATG18a* gene in heat stress

**DOI:** 10.3389/fpls.2024.1439972

**Published:** 2024-08-27

**Authors:** Xi Zhu, Wei Li, Ning Zhang, Huimin Duan, Hui Jin, Zhuo Chen, Shu Chen, Jiannan Zhou, Qihua Wang, Jinghua Tang, Yasir Majeed, Yu Zhang, Huaijun Si

**Affiliations:** ^1^ Key Laboratory of Tropical Fruit Biology, Ministry of Agriculture and Rural Affairs/Key Laboratory of Hainan Province for Postharvest Physiology and Technology of Tropical Horticultural Products, South Subtropical Crops Research Institute, Chinese Academy of Tropical Agricultural Sciences, Zhanjiang, Guangdong, China; ^2^ National Key Laboratory for Tropical Crop Breeding, Sanya Research Institute, Chinese Academy of Tropical Agricultural Sciences, Sanya, China; ^3^ State Key Laboratory of Aridland Crop Science, Gansu Agricultural University, Lanzhou, China; ^4^ College of Life Science and Technology, Gansu Agricultural University, Lanzhou, China; ^5^ College of Agronomy, Gansu Agricultural University, Lanzhou, China

**Keywords:** autophagy, potato, StATG18a, physiological, photosynthesis, heat stress

## Abstract

Autophagy is a highly conserved process in eukaryotes that is used to recycle the cellular components from the cytoplasm. It plays a crucial function in responding to both biotic and abiotic stress, as well as in the growth and development of plants. Autophagy-related genes (ATG) and their functions have been identified in numerous crop species. However, their specific tasks in potatoes (*Solanum tuberosum* L.), are still not well understood. This work is the first to identify and characterize the potato *StATG18* subfamily gene at the whole-genome level, resulting in a total of 6 potential *StATG18* subfamily genes. We analyzed the phylogenetic relationships, chromosome distribution and gene replication, conserved motifs and gene structure, interspecific collinearity relationship, and cis-regulatory elements of the ATG18 subfamily members using bioinformatics approaches. Furthermore, the quantitative real-time polymerase chain reaction (qRT-PCR) analysis suggested that *StATG18* subfamily genes exhibit differential expression in various tissues and organs of potato plants. When exposed to heat stress, their expression pattern was observed in the root, stem, and leaf. Based on a higher expression profile, the *StATG18a* gene was further analyzed under heat stress in potatoes. The subcellular localization analysis of StATG18a revealed its presence in both the cytoplasm and nucleus. In addition, *StATG18a* altered the growth indicators, physiological characteristics, and photosynthesis of potato plants under heat stresses. In conclusion, this work offers a thorough assessment of *StATG18* subfamily genes and provides essential recommendations for additional functional investigation of autophagy-associated genes in potato plants. Moreover, these results also contribute to our understanding of the potential mechanism and functional validation of the *StATG18a* gene’s persistent tolerance to heat stress in potato plants.

## 1 Introduction

The potato (*Solanum tuberosum* L.) has its origins in the Andes Mountains of South America and flourishes in the low-temperature climate. According to Zheng et al. (2024), it is the third most consumed and stable crop in the world, following rice and wheat. With its remarkable adaptability and a greater percentage of edible biomass (~85%) compared to cereals (~50%), it is one of the most significant crops in the world (Lutaladio and Castaldi, 2009). Depending on the cultivar and season, the potato crop can flourish in soils with a pH range of 5–7.5 and produce a yield of 40–70 tons per hectare (Fuqiang et al., 2021). This crop has a short growth season and may be grown in various environmental conditions (Muhammad and Zoltan, 2022). The majority of cultivated plant varieties are susceptible to heat. Temperature is considered the main uncontrolled factor that affects the development and output of potatoes (Zhang et al., 2024). The average day temperature range of 14–22°C is excellent for the growth of most commercial potato cultivars, deviating from this range significantly reduces the yield (Siano et al., 2024). The primary aspects of climate change are the greenhouse effect and global warming, which significantly impact on ecology, the agriculture industry, and food security at both national and international levels by affecting agricultural productivity. The greenhouse effect and global warming impact on potato production is anticipated to distress world yield by 18–32% by 2050 (Anderson et al., 2020). High temperatures have diverse effects on the growth and development of potatoes. They have the ability to reduce the total leaf area, increase the number of flowers per branch, restrict root development, promote stem growth, and boost branching (Tang et al., 2018). Elevated temperatures cause damage to chloroplasts, which overall lowers the amount of chlorophyll significantly. Consequently, the rate of photosynthetic respiration and assimilate synthesis decreases, causing a shift in the distribution of assimilate from tubers to leaves. Heat stress leads to decreased tuber induction, formation, development, and expansion (Singh et al., 2020). In addition, it induces necrosis, decreases dry matter content, and leads to tuber deformities (Rudack et al., 2017).

Autophagy—”self-eating”—is one of the numerous mechanisms and cellular responses in agricultural plants engaged under extreme climatic conditions. The process is a highly conserved system in eukaryotes that involves the breakdown and recycling of many cytoplasmic components, including damaged nuclear fragments, malfunctioning complexes, proteins, and even entire organelles (Lal et al., 2022). Plants have been observed to exhibit three separate but non-exclusive forms of autophagy: macro-autophagy, micro-autophagy, and mega-autophagy (Marshall and Vierstra, 2018). Macro-autophagy is the process in which cellular loads are sequestered and appropriated by the double-membrane structures of autophagosomes. These structures then join with the vacuole for digestion and recycling. It frequently leads to the random degradation of bulk proteins and the identification of autophagy substrates through specialized receptors, selectively eliminating particular components (Schuck, 2020; Luong et al., 2022). On the other hand, the transportation of anthocyanin aggregates from the cytosol to the vacuole is an instance of the direct absorption of cytoplasmic substances into the vacuole by the inward folding or outward bulging of the tonoplast during micro-autophagy. The vacuolar membrane directly engulfs these components, causing them to eventually be released into the vacuolar lumen (Rubio-Tomás et al., 2023), Mega-autophagy is an extreme form of autophagy that occurs when the vacuolar membrane becomes permeable or ruptures.

Previous studies demonstrated that several autophagy-related genes have been discovered in different crop plants. Recently, ATG homologs have been discovered in plants as well as animals, whereas yeast (*Saccharomyces cerevisiae*) is the very first organism in which the ATG homolog was discovered (Rehman et al., 2021). Besides, in many plant species number of ATG homologs have been identified, such as in model algae Chlamydomonas (*Chlamydomonas reinhardtii*), (Pérez-Pérez and Crespo, 2010), *Arabidopsis* (*Arabidopsis thaliana*) (Kwon et al., 2010), wheat (*Triticum aestivum*) (Cui et al., 2018), rice (*Oryza sativa*) (Marshall and Vierstra, 2018), maize (*Zea mays*) (Pérez-Pérez and Crespo, 2010), tobacco (*Nicotiana tabacum*) (Zhou et al., 2015) and tomato (*Solanum lycopersicum*) (Zhou et al., 2014). The up-regulation of ATG homologue in crop plants enhanced the autophagic flux, boosted seed yield, and fortified deferred aging (Brimson et al., 2021). The expression of *ATG* genes in plants’ response to abiotic stress may be controlled at the transcriptional level by certain transcription factors. Such as heat shock transcription factor SlHsfA1a (Wang et al., 2015), ethylene response factor SlERF5 (Zhu et al., 2018) and transcription factor SlWRKY33a/SlWRKY33b (Zhou et al., 2014) in tomato. Moreover, it has been shown that genes responsible for transcription factors play crucial roles in the response of other plants to abiotic stress, except for model plants and crops (Li et al., 2022b; Han et al., 2018, Han et al., 2020, Han et al., 2021; Han et al., 2023). Autophagy-related genes have been extensively investigated in many crops to understand their involvement in mitigating heat stress. Studies have shown that autophagy has a crucial role in alleviating heat stress, leading to the accumulation of autophagosomes in tomatoes, apples, and *Arabidopsis* (Kwon et al., 2010; Zhou et al., 2014; Huo et al., 2020). There was a suggestion that heat stress causes endoplasmic reticulum (ER) stress, which then triggers autophagy. ER stress occurs due to the buildup of unfolded proteins in the endoplasmic reticulum, resulting in the creation of protein aggregates (Yang et al., 2016). The *Arabidopsis AtATG5* and *AtATG7* genes displayed greater susceptibility to heat stress compared to wild-type plants, as seen by heightened wilting, increased electrolyte leakage, and reduced photosynthetic performance. Furthermore, the *AtATG7* gene exhibited an accumulation of insoluble protein aggregates that were tagged with ubiquitin (Zhou et al., 2013). Similarly, Zhou et al. (2014) conducted a study, which revealed that when tomatoes were subjected to heat stress, they observed comparable results in the virus-induced gene silencing (VIGS) of *SlATG5* and *SlATG7*. The temperature is progressively rising due to climate change, mostly caused by the greenhouse effect and global warming. Heat stress impacts all stages of crop plants and immediately decreases the yield and productivity. Developing heat-tolerant crop varieties is imperative to meet the rising food requirements of the expanding population amidst the challenges presented by climate change. This is a novel investigation that identified the autophagy-related genes of subfamily *StATG18* in potato plants on the base of genome-wide identification through bioinformatics analysis. A comprehensive search approach included 6 putative *StATG18* subfamily genes comprising *StATG18a, StATG18b, StATG18c, StATG18d, StATG18f*, and *StATG18h*.

To further understand the functional, structural, and evolutionary characteristics of potato *StATG18* subfamily genes, we analyzed the phylogenetic relationship, chromosomal distribution, and gene duplication, analysis of conserved motifs and gene structure, interspecific collinearity relationship, and cis-regulatory elements in potatoes. An extensive investigation was carried out on differential expression analysis of the *StATG18* subfamily gene in various tissues and organs such as tuber, flower, petiole, stem, stolon, leaf, and root of potato plants and then under heat stress, differential expression analysis of roots, stems, and leaves of potato plants were also performed by using qRT-PCR analysis. Additionally, functional validation of the novel gene *StATG18a* response to heat stress will be provided in this article. This article will also provide a fresh perspective for researchers to understand the molecular, physiological, and biochemical processes of the *StATG18a* gene in potato plants under heat stress.

## 2 Materials and methods

### 2.1 Identification of *StATG18* subfamily genes in potato plants

The phylogenetic tree of potato *StATG18* subfamily genes with other species such as *Arabidopsis*, rice, and tomato has been constructed as shown in **Supplementary Figure 1**. It was discovered that the nucleotide sequences of the *StATG18* subfamily genes may be used to find the genes inside the potato genome using the BLAST database of potatoes (http://spuddb.uga.edu/blast.shtml). Similarly, another approach included using the term “autophagy” to search the potato genome database (http://spuddb.uga.edu/integrated_searches.shtml) to identify genes belonging to the StATG18 subfamily. Following the removal of unnecessary genes, a database of the NCBI conserved domain (https://www.ncbi.nlm.nih.gov/Structure/bwrpsb/bwrpsb.cgi) was used to search all possible potato *StATG18* subfamily genes for autophagy-related domains (Marchler-Bauer et al., 2014). (The Spud DB potato genome database was used to ascertain the chromosomal location, coding sequence (CDS), and genome length of the predicted *StATG18* subfamily genes. The ExPasy website (https://web.expasy.org/protparam/), which provides the ProtParam software (Gasteiger et al., 2003), (was utilized to predict and identify the StATG18 subfamily proteins, amino acid count, theoretical molecular weight (MW), isoelectric point (pI), and grand average of hydropathicity (GRAVY) as shown in **Supplementary Table 1**. The subcellular localization of *StATG18* subfamily genes was predicted using the Plant-mPLoc website (http://www.csbio.sjtu.edu.cn/bioinf/plant/), as described by Chou and Shen, 2010.

### 2.2 Multiple sequence alignments and phylogenetic analysis

The protein files of potato (DM v4.03/v4.04), *Arabidopsis thaliana* (TAIR10), rice (*Oryza sativa* v7.0), and tomato (ITAG4.0) were obtained by downloading them from the Rice Genome Annotation Project database, Phytozome v13 database, TAIR database, and Spud DB potato genome database (http://spuddb.uga.edu/). The StATG18 subfamily proteins were found to have multiple sequence alignment (MUSCLE) (Edgar, 2004). With 1,000 Bootstrap repetitions and other default settings (phylogenetic reconstruction, model/method P-distance, alternative types: amino acids, inter-site ratio uniform ratio, full omission for gap deletion data processing), MEGA-X was used to create a phylogenetic tree (**Supplementary Figure 1**) (Dereeper et al., 2010), using the neighbor-joining (NJ) method.

### 2.3 Chromosome localization and gene duplication analysis

The Circos software (http://circos.ca/software/download/) was utilized to identify a total of 6 *StATG18* subfamily genes on potato chromosomes (Krzywinski et al., 2009). The physical position of the chromosomes was determined using the Spud DB Potato Genome database (http://spuddb.uga.edu/). The specific genes and their locations may be found in **Supplementary Table 1** and **Supplementary Figure 2**. Duplicate occurrences in *StATG18* subfamily genes were revealed by the default parameter analysis of adopting MCScanX (Multiple collinear scanning toolkit, MCScanX) (https://github.com/wyp1125/MCScanx) (Wang et al., 2012). The TBtools software (https://github.com/CJ-Chen/TBtools/releases) premeditates both non-synonym substitution (Ka) and synonym replacement (Ks), according to Chen et al. (2020). Emanuelsson et al. (2000) calculation formula was used to evaluate the divergence time of duplicated *StATG18* subfamily genes (**Supplementary Table 2** and **Supplementary Figure 2**).

### 2.4 Conserved motifs and gene structure analysis

The Multiple “Em for Motif Elicitation” (MEME) tool v5.3.0 (http://meme-suite.org/meme-software/5.3.0/meme-5.3.0.tar.gz) was used to identify the conserved motifs of the putative StATG18 protein (Li et al., 2022a), (**Supplementary Figure 3**, **Supplementary Table 3**). The following were the established parameters: ten motifs were analyzed, ranging in length from six to two hundred residues. To further annotate all motifs, Inter Pro Scan (http://www.ebi.ac.uk/interpro/) was used (Blum et al., 2021), as shown in **Supplementary Figure 4**. Six *StATG18* subfamily genes genome sequences and CDS were taken from the potato genome database Spud DB, Using the gene structure display server (GSDS 2.0) (http://gsds.gao-lab.org/) (Hu et al., 2015). The CDS of *StATG18* subfamily genes were connected to the pertinent genomic DNA sequence. Consequently, the distribution of exons and introns in *StATG18* subfamily genes was also determined, as seen in **Supplementary Figure 4**.

### 2.5 Synteny analysis of *StATG18* family genes

The protein sequences of rice, potato, *Arabidopsis*, and tomato were absorbed using the previously described method (**Supplementary Figure 5**). A source of tomato protein sequence data was obtained by Phytozome v13 (https://phytozome-next.jgi.doe.gov/). Using the Makeblastdb program, the protein sequences of the *StATG18* subfamily genes of these four plants were assembled into a local database (Johnson et al., 2008). The protein sequences of the potato and the other three plants were then coherent using the Blastp software (Jacob et al., 2008). The linear connection was assembled using the MCScanX software (https://github.com/wyp1125/MCScanx) (Wang et al., 2012).

### 2.6 Cis-elements in the promoter of *StATG18* family genes

The Perl programming language retrieved the 2000 bps DNA sequence of the 5ˊ end non-coding region upstream of the *StATG18* subfamily genes with the start codon “ATG”. Next, download the promoter region sequence using PlantCARE (http://bioinformatics.psb.ugent.be/webtools/plantcare/html/), examine each binding site, and forecast its possible function in the cis-regulatory elements displayed in **Supplementary Table 4**, **Supplementary Figure 6** (Lescot et al., 2002).

### 2.7 Plant growth and heat treatment

In this study, the potato (*Solanum tuberosum* L.) variety ‘Atlantic’ (pH 5.8–6.0) was cultivated on MS medium containing 3% sucrose and 0.7% agar. A Biotron was used for the incubation, which had a temperature of 22°C, an 8-hour dark cycle, and a 16-hour photoperiod (2800 Lx). After four weeks, they were grown on MS media and given heat treatment (35°C) at different time intervals, such as 0 (control) 1, 2, 4, 8, 16, 24, and 48 h for three biological replicates and three technical replicates. Leaves stems, and roots of harvested plants were stored in liquid nitrogen at -80°C, and the *StATG18* subfamily genes were detected by quantitative reverse transcription-PCR (qRT-PCR). The sprouting tubers were grown to a developmental stage that allowed for the collection of leaves, flowers, petioles, stems, stolons, and roots to determine the differences in *StATG18* subfamily gene expressions across different tissues. The tubers were then gathered until they attained the mature stage. The samples were frozen in liquid nitrogen at -80°C before analyzing the relative expression level of *StATG18* subfamily genes (*StATG18a*, *StATG18b*, *StATG18c*, *StATG18d*, *StATG18f* and *StATG18*) by qRT-PCR.

### 2.8 RNA extraction and qRT-PCR analysis

Total RNA was extracted from the obtained samples using the TRIzol RNA Extraction kit (Invitrogen, Carlsbad, CA, USA). Using the First-Strand cDNA Synthesis Kit (TransGen Biotech, Beijing, China), the target genes’ first-strand cDNA was created. A LightCycler 480 II real-time PCR machine (Roche, Rotkreuz, Switzerland) was utilized to perform quantitative polymerase chain reaction (qPCR). The reaction mixture consisted of 0.8 μL of 0.5 μM specific primers, 100 ng of cDNA, and 10 μL of SYBR Premix Ex Taq (2 ×) (Takara, Tokyo, Japan). After 3 min of initial incubation at 94°C, the reactions were subjected to 36 cycles of 94°C for 45 s, 59°C for 34 s, and 72°C for 1 min. The *Stef1a* was used as a reference gene for standardization, and relative expression was calculated using the 2^−ΔΔCT^ method (Livak and Schmittgen, 2001). **Supplementary Table 5** provides a list of primer sequences used in this study.

### 2.9 Construction and transformation of the plasmid

To create overexpressing *StATG18a* plants (OE plants), the encoding sequence of *StATG18a* was cloned into the pBI121-EGFP plasmid using a previously published method (Li et al., 2020). Transformation studies were conducted using *Agrobacterium tumefaciens* strains LBA4404 for overexpression. Using the previously reported method, potato plants were created to knockdown the expression of StATG*18a* by RNA interference (Ri plants) (Lu et al., 2019). **Supplementary Table 5** provides a list of primer sequences used in this study. After being cultivated for about 48 hours at 28°C in LB medium with 50 mg of gentamicin and 50 mg of spectinomycin, plasmid-containing agrobacterium was recovered by centrifugation (5,000 rpm, 10 min) and re-suspended in MS medium (OD600 = 0.3). After being cultivated in Agrobacterium solution for 10 min, the sterile seedling stems (2 cm) were grown in MS medium (pH: 5.8) that included 7.4 g/L agar, 30 g/L sucrose, 0.5 mg/L 6-BA, 2.0 mg/L ZT, 0.2 mg/L GA3, and 1.0 mg/L IAA. The growth medium was then kept in the dark for 48–72 hours. The seedlings were then placed in a differentiation medium (MS, 7.4 g/L agar, 30 g/L sucrose, 300 mg/L Timentin, 100 mg/L kan, 0.5 mg/L 6-BA, 2.0 mg/L ZT, 0.2 mg/L GA3, and 1.0 mg/L IAA; PH: 5.8), and changed every two weeks. Once adventitious roots were induced, the resistant adventitious buds were moved to a root media that had been prepared (MS + 7.4 g/L agar + 30 g/L sucrose + 300 mg/L Timentin + 100 mg/L kan, pH=5.8).

### 2.10 Subcellular localization of the StATG18a protein

To find out the subcellular localization of the StATG18a protein in potato plants, the *StATG18a* (XM_006356959.2) was amplified using the primers (pBI121-EGFP), with forward primer (5’-ATGGCCACTGTTTCCCCTCTCC-3’) and reverse primer (5’-CGAGGCCTTTTCGGACTTC AGA-3’) sequences. After being ligated into the pBI121-EGFP vector and mediated by the *Agrobacterium tumefaciens* strain (Gv3101), the recombinant plasmid pBI121-EGFP-StATG18a was created. The total RNA was then extracted for PCR examination. Tobacco epidermal cells are injected with the recombinant plasmid Gv3101 (Sparkes et al., 2006). These plants were maintained at 24°C for 12 hours in a dark greenhouse. After being treated for 48 hours, the plant’s epidermal cells were extracted, and the stained piece was then cut apart. Leica TCA confocal scanning laser microscope (Leica, Weztlar, Germany) was used to detect the green fluorescent protein signals.

### 2.11 Phenotypic analysis between transgenic and non-transgenic (NT) potato plants

Again four-weeks-old seedlings of transfected and non-transfected potato plants were grown in MS media containing 8% sucrose. The seedlings were cultivated for 30 d to create potato tubers, and then the sprouting tubers were cultivated in pots (26.2 cm diameter, 24.8 cm height) supplemented with nutrition soil and vermiculite (1:1, v/v). To determine the growth indexes (plant height, total fresh and dry weights, as well as fresh and dry root weights) transgenic and non-transgenic potato plants were grown in pots for 6 weeks as a control, then potted plants (transgenic and non-transgenic) were grown for another six-weeks and subjected to heat stress for 48 hours before returning to normal temperature for 7 d. Similarly, again potted plants (transgenic and non-transgenic) were grown for another six weeks subjected to heat stress for 48 hours, and returned to normal temperature growth state for 14 d. The point where the soil surfaces meet the apex of the shoot was used to measure the height of the plant. The biomass was calculated by weighing all of the plant’s fresh and dried components together. The entire plant was maintained in an oven at 70°C until consistent bulks were reached in order to determine the dry weight. In this experiment, there was a complete random disturbance of the triplicated treatments.

### 2.12 Determination of physiological and biochemical indicators

Four-week-old transgenic and non-transgenic potato plantlets were cultivated in MS media supplemented with 8% sucrose to encourage tuber formation. The sprouting tubers were cultivated in pots for six weeks with soil water content in the pots was 70–75%, soil water content was monitored at 10:00 and 16:00 every day using TDR-300 sensors (Spectrum R, Aurora, IL, USA). The leaves were subjected to heat treatment at different time intervals (0, 4, 8, 16, 24, and 48 h), after which they were collected and measured using methods previously reported for proline (Bates et al., 1973), malondialdehyde (MDA) (Heath and Packer, 1968) and hydrogen peroxide (H_2_O_2_) (Bouaziz et al., 2015), as well as superoxide dismutase (SOD), catalase (CAT), and peroxidase (POD) (Li, 2000). Each treatment was arranged in triplicate with a completely random configuration. A total of 270 pots (5 lines × 6 treatments × 3 replicates × 3 pots) were used in each repeat, with 3 pots per treatment and 1 plant per pot.

### 2.13 Measurements of gas exchange parameters

The third leaf from the top of the plant was detected between 9:30 and 11:30. The stomatal conductance, transpiration rate, and net photosynthetic rate were measured using a portable photosynthetic LI-6400XT system (Li-COR, Lincoln, NE, USA). A fixed photon flux density of 1500 μmol·m^−2^·s^−1^ was used. Relative humidity in the leaf chamber ranged from 60 to 70 percent. There was 400 μmol/mol of CO_2_. Each treatment was set up in triplicate using a fully random design. There were 3 pots and 1 plant per pot in each repeat for every treatment, for a total of 270 pots (5 lines × 6 treatments × 3 replicates × 3 pots).

### 2.14 Statistical analysis

All statistical analyses were done using IBM SPSS 19.0 Statistical Software (IBM, Chicago, IL, USA) and GraphPad Prism Software (GraphPad, San Diego, CA, USA). The findings are shown as mean plus standard deviation. The program GraphPad Prism was used to design line charts and histograms. One-way ANOVA with the Tukey test, Dunnett’s T3 for posthoc analysis, or two-way ANOVA adjusted by Sidak’s multiple comparisons test was used for multiple comparisons.

## 3 Results

### 3.1 Genome-wide identification of the *StATG18* subfamily genes in potato

The availability of the nucleotide sequence facilitated the identification of all the genes belonging to the *StATG18* subfamily in potatoes. After a thorough search and validation of the entire potato genome, a total of 6 potential *StATG18* subfamily genes were identified. Members of the *STATG18* gene subfamily in potato plants were identified and classified, and **Supplementary Table 1** displayed the physicochemical properties and characteristics of *STATG18* subfamily genes found in the potato genome. Using the Spud DB potato genome database, the anticipated chromosomal position, CDS, and genome length of the *StATG18* subfamily genes were determined. **Supplementary Table 1** provided the following information, which was utilized to assess the identified *StATG18* subfamily genes: protein ID, gene ID, chromosomal position, strand type, amino acid length (AAL),MW, pI, GRAVY and subcellular localization. The maximum MW was given for *StATG18h* (95453.87), amino acid length (878), while the minimum was given for *StATG18b* (39707.58), amino acid length (365). Similarly, the maximum pI was observed for *StATG18f*a (8.44), whereas the minimum was 6.3 for *StATG18h.* The maximum positive value for GRAVY was calculated for *StATG18b* (0.13) and the minimum negative values amounted to *StATG18f* (-0.283). The subcellular localization described that the *StATG18a* is localized in the cytoplasm as well as in the nucleus, *StATG18b, StATG18c*, and *StATG18d* are located only in the nucleus, while *StATG18f* is located in the nucleus as well as chloroplast and *StATG18h* is only located only in the chloroplast.

### 3.2 Polygenetic analysis of *StATG18* subfamily members in potato

An evolutionary tree was generated to analyze the phylogenetic relationship among members of the *StATG18* subfamily in potatoes and other species, including *Arabidopsis*, rice, and tomato, as depicted in **Supplementary Figure 1**. Phylogenetic analysis revealed that the StATG18 subfamily protein sequences were extremely comparable to their homologues (*Arabidopsis*, rice, and tomato) and it is evident from the observation that many internal branches have maximum bootstrap values that pairs of potentially orthologue proteins with comparable functions have been derived from a common ancestor in a statistically valid manner. The phylogenetic tree also showed that the potato *StATG18* subfamily genes were more closely related to tomatoes. The *StATG18* subfamily genes in potatoes (*StATG18a*, *StATG18b*, *StATG18c*, *StATG18f*, and *StATG18h*) have a one-to-one correspondence with their tomato counterparts (*SlATG18a*, *SlATG18b*, *SlATG18c*, *SlATG18f*, and *SlATG18h*), as seen in **Supplementary Figure 1** with bootstrap values of 100. These proteins may play similar functions *in vivo*.

### 3.3 Chromosomal localization and gene replication analysis of *StATG18* subfamily genes

The physical map positions of the 6 *StATG18* subfamily genes on 12 chromosomes of the potatoes were displayed in **Supplementary Figure 2**. The distribution of these *StATG18* subfamily gene homologs throughout the chromosomes appeared to be irregular. The *StATG18* subfamily genes, such as *StATG18a* and *StATG18d* are located on chromosomes 8, *StATG18b* and *StATG18h* are situated on chromosome 7, *StATG18c* is positioned on chromosome 1, and *StATG18f* is found on chromosome 12. The duplicate chromosomal regions at different lengths were displayed by the red lines in **Supplementary Figure 2**. The red line connected the duplicate chromosomal portions on chromosome 8, which connected the *StATG18a* gene with *StATG18d.* Additionally, The Ka/Ks ratio is a useful indication for understanding the process of gene differentiation after replication. The *StATG18* subfamily genes, *StATG18a/StATG18d* have a Ka/Ks ratio of less than 1, indicating that these duplicates were selected for the following purification. Moreover, no replicator pair greater than one is found, indicating that positively selected replicators are absent from the potato *StATG18* subfamily genes. **Supplementary Table 2** computes the time needed to replicate the event. The previous 17.76 million years of replication occurrences for the pair *StATG18a*/*StATG18d* were detected. As indicated in **Supplementary Table 2**, a Ka/Ks analysis and an estimation of the absolute dates for the duplication events between the duplicated *StATG18* subfamily genes in potatoes were noted.

### 3.4 Conserved motifs and gene structure analysis

A tool called MEME motif was utilized to perform conservative motif analysis of ATG18 subfamily proteins in potato, *Arabidopsis*, rice, and tomato, based on their amino acid sequences. Within *StATG18* subfamily members, the conserved motifs are numbered from 1 to 10. The highest number of conservative motifs were found in the *StATG18a* (7), *StATG18c* (7), and *StATG18d* (7) and minimum numbers were found in *StATG18b* (4), while the rest of the *StATG18* subfamily members have 5 numbers of conservative motifs shown in **Supplementary Figure 3** and **Supplementary Table 4**. These analyses revealed that all StATG18 subfamily members in potato have similar motif composition to ATG18 subfamily members in *Arabidopsis*, rice, and tomato, with a high degree of homology. Similar amino acid conserved domain composition suggests that similar gene functions may exist. The distribution of these conserved motifs also indicates that *StATG18* subfamily genes are relatively conserved in the evolutionary process.

In comparing the exon-intron architectures of potato *StATG18* subfamily genes to those of *Arabidopsis*, rice, and tomato, it was found that the most of *StATG18* subfamily genes exhibited greater conservatism in terms of exon length and number. Additionally, a noticeable variation in exon-intron structure was seen when compared to those of *Arabidopsis* and rice. To investigate the structural variety of *StATG18* subfamily gene sequences, the quantity and length of introns and exons in *StATG18* subfamily genes were also measured. The 6 *StATG18* subfamily genes are categorized and compared to the ATG18 subfamily genes of rice, tomato, and *Arabidopsis*, as seen in **Supplementary Figure 4**. Furthermore, the exon numbers varied in each member of subfamily *StATG18*, such as *StATG18a*, and *StATG18d* containing 4 exons, *StATG18f*, and *StATG18h* have 7 exons, and *StATG18b* has 11 exons. The intron length of *StATG18* subfamily genes was given as *StATG18a* (3.8 Kb)*, StATG18b* (4.8 Kb)*, StATG18c* (2.6 Kb)*, StATG18d* (2.7 Kb)*, StATG18f* (1.9 Kb), and *StATG18h* (4.5 Kb). Certain *StATG18* subfamily genes revealed similarities in the length and number of exon-intron compositions with other plant species, which suggests that the potato, *Arabidopsis*, rice, and tomato ATG18 subfamily genes may be consistent in specific functions.

### 3.5 Synteny analysis and identification of cis-regulatory elements of *ATG18* subfamily members in potato

The study of gene evolution can offer a more thorough understanding of the roles of *StATG18* subfamily genes. As **Supplementary Figure 5** illustrates a comparative synteny map among the genomes of potatoes, *Arabidopsis*, rice, and tomatoes. Three orthologous genes were identified in *StATG18* and *AtATG18* subfamily members (**Supplementary Figure 5**). The chromosomes 1 of *Arabidopsis* and potato contain an orthologous gene (PGSC0003DMT400063510/AT1G54710.1), whereas chromosome 4 of *Arabidopsis thaliana* and chromosome 7 of potato contain an orthologous gene (PGSC0003DMT400028996/AT4G30510.1). There was a collinear relationship among *AtATG18f* (AT5G5 4730.1), *StATG18f* (PGSC0003DMT400065820) and *StATG18h* (PGSC0003DMT400049486) on chromosome 5 of *Arabidopsis Thaliana*. Three gene pairs in rice have a collinear relationship with potatoes. There is also a pair of orthologous genes on chromosome 1 of each rice and potato (PGSC0003DMT400063510/LOC_Os01g70780). A pair of orthologous genes between chromosome 1 of rice and chromosome 7 of potato (PGSC0003DMT400049486/LOC_Os01g57720) was also detected. Similarly, chromosome 2 of rice and chromosome 7 of potato also have a pair of orthologous genes (PGSC0003DMT400028996/LOC_Os02g54910). However, as shown in **Supplementary Figure 5**, the *ATG18* family members in tomatoes are collinear in all *ATG18* family members in potatoes.

To investigate the biological functions and regulatory networks of *StATG18* subfamily genes in potatoes, we obtained the promoter region sequence located 2000 bp upstream of these genes. We then analyzed the cis-acting elements using Plantcare website and identified 37 cis-acting elements. Most of them have roles linked to light responsiveness, hormones, metabolism, regulation, and stress. More specifically, eight light-responsive modules, four MYB binding sites, four components related to various stressors (cold, drought, and wound), three auxin-responsive elements, two gibberellin-responsive elements, one MeJA-responsive element, one salicylic and abscisic acid component each, and some cis-elements were involved in regulation as the metabolism of various substances were linked to the hormone-response elements. Furthermore, as **Supplementary Figure 6** illustrates most cis-regulatory elements were specifically associated with endosperm expression, anaerobic induction, regulation of zein metabolism, differentiation of palisade mesophyll cells, circadian control, regulation of the cell cycle, flavonoid biosynthesis, and regulation that is specific to seeds. According to these results, *StATG18* subfamily genes could be involved in metabolic regulation, biochemical stimulation, growth and development of potatoes, hormone signaling transduction, and the regulation of different stress responses. Number statistics of different 37 cis-regulatory elements in promoter regions of potatoes for different genes (*StATG18a*, *StATG18b*, *StATG18c*, *StATG18d*, *StATG18f*, and *StATG18h*) of subfamily *StATG18* were given in **Supplementary Table 5**. In particular, the majority of the genes were found to be involved in metabolic control, hormone stimulation, light-responsiveness, stress-responsiveness, and metabolism, all of which define the important roles that *StATG18* subfamily genes play in signal transduction and evolutionary processes.

### 3.6 Different *StATG18* subfamily genes were recognized as differently expressed in various tissues and organs of potato plants under heat stress.

**Figure 1:**
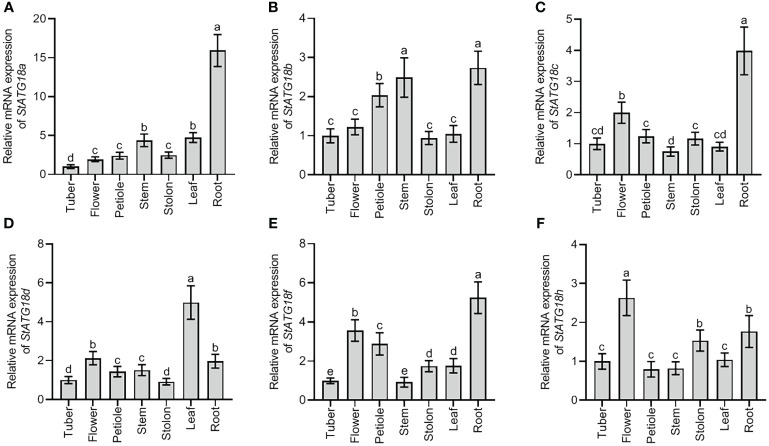
Expression profiles of *StATG18* subfamily genes in different tissues of potato. Transcript levels of **(A)** *StATG18a*, **(B)** *StATG18b*, **(C)** *StATG18c*, **(D)** *StATG18d*, **(E)** *StATG18f*, and **(F)** *StATG18h* at mRNA levels, in the tuber, flower, root, petiole, stem, leaf and root. Data were the means ± standard deviation. Different letters indicated significant differences between the two groups (*p*-value less than 0.05, calculated by one-way ANOVA, followed by LSD and Duncan or Dunnett’s T3).

**Figure 2:**
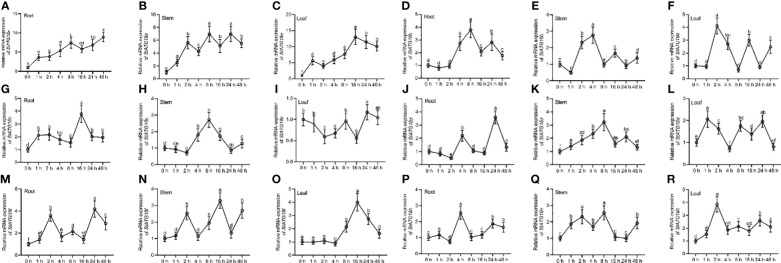
Expression profiles of *StATG18* subfamily genes in potato leaves, stem and roots in response to heat stress. Transcript levels of **(A–C)** *StATG18a*, **(D–F)** *StATG18b*, **(G–I)** *StATG18c*, **(J–L)** *StATG18d*, **(M–O)** *StATG18f* and **(P–R)** *StATG18h* in leaves, stems, and roots under heat stress treatment at different time intervals (0, 1, 2, 4, 8, 16, 24, and 48h). Data were the means ± standard deviation. Different letters indicated significant difference between two groups (*p*-value less than 0.05, calculated by one-way ANOVA, followed by LSD and Duncan or Dunnett’s T3).

Using qRT-PCR analysis, the relative mRNA expression pattern showed distinct *StATG18* subfamily genes that were found in potato plants’ tuber, flower, petiole, stem, stolon, leaf, and root, as shown in **Figures 1A–F**. The highest level of expression was seen in the roots for *StATG18a*, *StATG18b*, *StATG18c*, and *StATG18f*, while *StATG18d* showed the highest expression in the leaf, and *StATG18h* presented the highest expression in the flower, as shown in **Figure 1**. In addition, the *StATG18b* and *StATG18f* genes showed higher levels of expression in the stem and petiole, and in the flower and petiole, respectively. The findings suggest that the *StATG18* subfamily genes have a role in the growth and development of different tissues and organs in potatoes. Additionally, we inspected the relative mRNA expression profile of *StATG18* subfamily genes (*StATG18a, StATG18b, StATG18c, StATG18d, StATG18f*, and *StATG18h*) in roots, stems and leaves of potato plants under heat stress at different time intervals (0, 1, 2, 4, 8, 16, 24and 48 h), while (0 h) was considered as control group. Results from qRT-PCR analysis exhibited that the *StATG18a* in roots, stems, and leaves were up-regulated (*p* < 0.05) after heat treatment. In the same way, the rest of the *StATG18* subfamily genes (*StATG18b, StATG18c, StATG18d, StATG18f*, and *StATG18h*) showed up-regulation first and then down-regulation expression in roots, stems, and leaves after heat treatment at different time intervals showed in **Figure 2**.

### 3.7 Subcellular localization analysis of StATG18a

**Figure 3:**
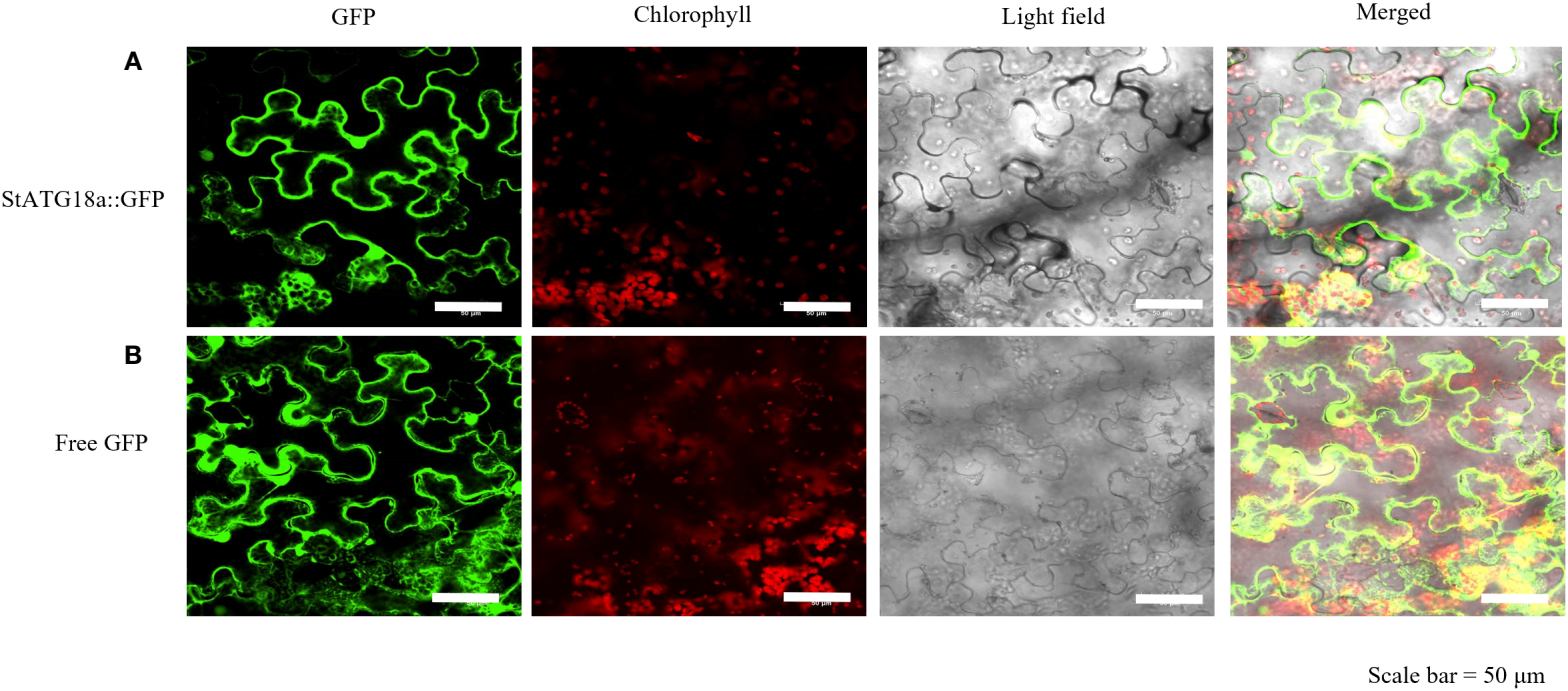
Subcellular localization of StATG18a-GFP fusion protein in *Nicotiana benthamiana* leaves. **(A)** Confocal scanning laser microscopy analysis of tobacco transformed with pBI121-EGFP-StATG18a. **(B)** The empty vector (GFP) served as a control. Bar = 50 μm.

The protein localization of StATG18a was predicted using the Subcellular Plant-mPLoc website (http://www.csbio.sjtu.edu.cn/bioinf/plant-multi/), which predicted that the StATG18a protein is located in the nucleus. To govern the subcellular localization of StATG18a, The recombinant plasmid pBI121-EGFP-StATG18a was transformed into *Agrobacterium tumefaciens* GV3101 and then transiently transformed into tobacco. The green fluorescence from the EGFP vector expressed by pBI121-EGFP-StATG18a demonstrated that the StATG18a was observed in the cytoplasm as well as in the nucleus by confocal-scanned microscopy as shown in **Figure 3**. To confirm the localization of StATG18a, the EGFP empty vector or control vector was presented in the membrane, cytoplasm, and nucleus, while the pBI121-EGFP-StATG18a was detected in the cytoplasm and nucleus, which confirmed the presence of StATG18a in potato plants. Despite the *StATG18a* being discovered in potato plants, its role has not been specified to date, which is needed for further analyses under heat stress conditions.

### 3.8 *StATG18a* was involved in modulating potato growth

**Figure 4:**
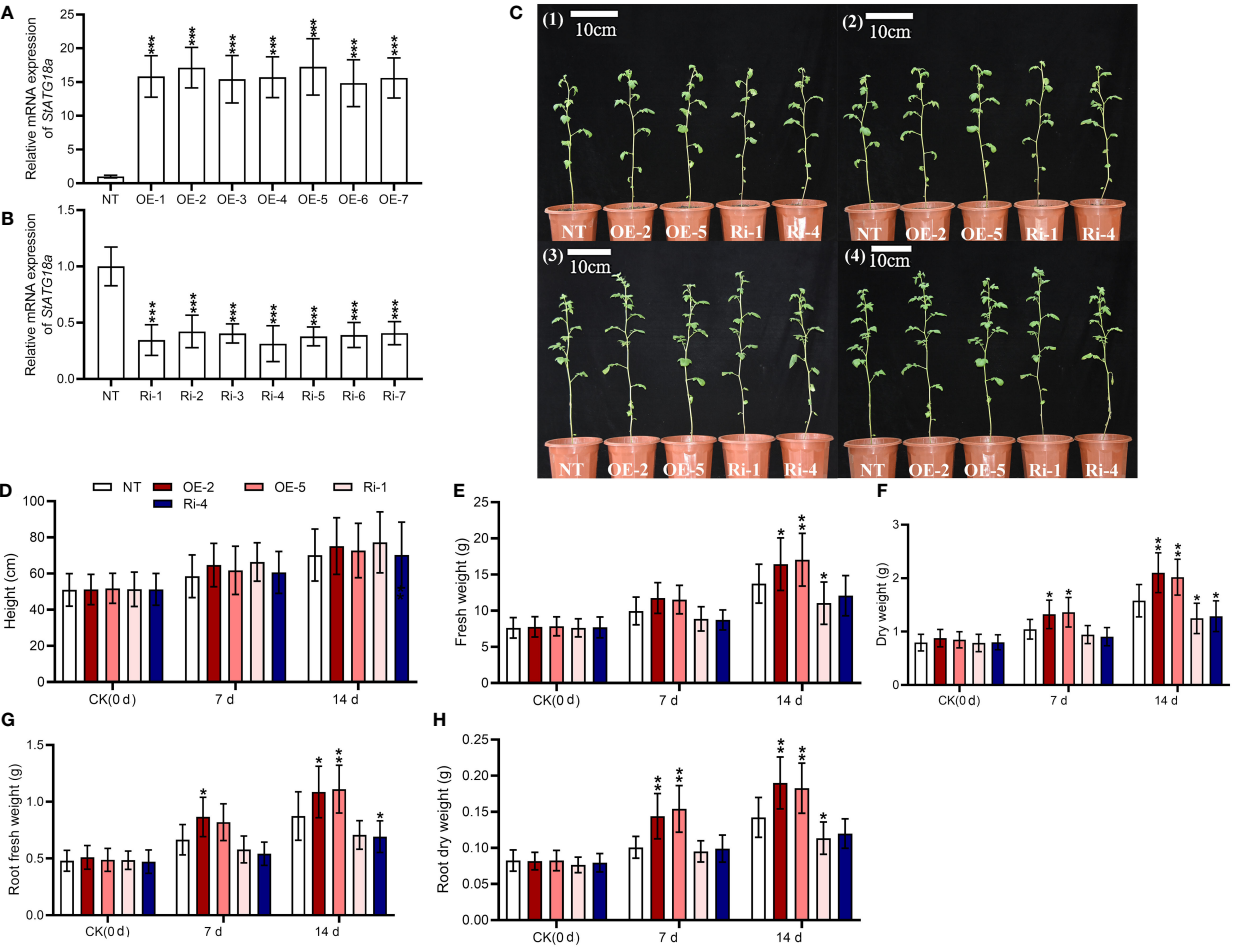
Effects of *StATG18a* on potato plant growth phenotypes. Transcript levels of *StATG18a* in **(A)** overexpressing *StATG18a* and **(B)** knock down potato plants (*p < 0.05, **p < 0.01, ***p< 0.001). One-Way ANOVA corrected by Dunnett (**Figures 5A, B**, n = 9). **(C1)** The phenotypes of transgenic and non-transgenic potato plants were grown normally for 6 weeks, **(C2)** The phenotypes of transgenic and non-transgenic potato plants treated with 35°C heat stress for 48 hours after 6 weeks of normal growth, **(C3)** The phenotypes of transgenic and non-transgenic potato plants that recovered 7 d after heat stress treatment, **(C4)** The phenotypes of transgenic and non-transgenic potato plants that recovered 14 d after heat stress treatment, and **(D)** plant height, **(E)** total fresh weights and **(F)** dry weights, as well as **(G)** root fresh weights and **(H)** dry weights were measured after the heat treatment at different time intervals (0 d, 7 d and 14 d), Bar = 10 cm. NT, non-transgenic plants; OE, pBI121-EGFP-StATG18a-transgenic plants (OE-2 and OE-5); Ri, pART-StATG18a-RNAi-transgenic plants (Ri-1 and Ri-4); Two-Way ANOVA amended by Tukey (**Figures 4D–H**, n = 9; *p < 0.05, **p< 0.01).

We created overexpressing *StATG18a* and knockdown potato plants to examine the effects of *StATG18a* on potato growth under heat stress. After screening stably transformed plants, their expression efficiency was confirmed by RT-qRCR, as seen in **Figures 4A, B** (***p < 0.001). We observed the phenotypes of transgenic and non-transgenic plants under normal and heat-stress conditions. Phenotypic analysis of transgenic and non-transgenic potato plants showed no significant difference under control conditions (**Figure 4C1**). After 48 hours of heat stress treatment, there was still no significant difference in phenotype, as shown in **Figure 4C2**. However, on the 7th and 14th d after heat treatment, we found that some leaves of non-transgenic and *StATG18a* knockdown plants turned yellow and began to wilt, while the leaves of overexpressing *StATG18a* transgenic plants remained normal and did not wilt, as shown in **Figures 4C3, C4**. There were no statistically significant changes in plant height for all potato plants after 7 and 14 d of heat stress treatment. However, transgenic potato plants (OE-2, OE-5, Ri-1, and Ri-4) showed significant differences in total fresh and dry weights, as well as root fresh and dry weights, as shown in **Figures 4D–H**. 7 d after heat treatment, the total fresh weight of overexpression plants (OE-2 and OE-5) exhibited an increase of 1.17 and 1.15 folds, respectively, compared to non-transgenic plants. In contrast, the total fresh weight of RNA interference-expressing plants (Ri-1 and Ri-4) decreased by 11% and 13%, respectively, when compared to the control group. Similarly, 14 d after heat treatment, the total fresh weight of overexpression plants (OE-2 and OE-5) showed an increase of 1.19-fold and 1.24-fold, respectively, relative to non-transgenic plants. Concurrently, the total of fresh weight of RNA interference-expressing plants (Ri-1 and Ri-4) was reduced by 20% and 12%, respectively, compared to the control group. On the 7th d after the heat treatment period, the root fresh weight of overexpression plants (OE-2 and OE-5) was found to be 1.3-fold and 1.23-fold greater than that of non-transgenic plants, respectively. Conversely, the root fresh weight of RNA interference-expressing plants (Ri-1 and Ri-4) was diminished by 13% and 19%, respectively, in comparison to non-transgenic plants. Likewise, on the 14th d after the heat treatment period, the root fresh weight of overexpression plants (OE-2 and OE-5) was observed to be increased by 1.24-fold and 1.27-fold, respectively, relative to non-transgenic plants. However, the root fresh weight of RNA interference-expressing plants (Ri-1 and Ri-4) was reduced by 19% and 21%, respectively, when compared to non-transgenic plants. Furthermore, 7 d after heat treatment, the dry weight of overexpression plants (OE-2 and OE-5) was found to be increased by 26% and 30%, respectively, in comparison to non-transgenic plants. In contrast, the dry weight of RNA interference-expressing plants (Ri-1 and Ri-4) was decreased by 10% and 14%, respectively, relative to the non-transgenic lines. Besides, 14 d after heat treatment, the dry weight of overexpressing *StATG18a* plants (OE-2 and OE-5) was increased by 1.33 and 1.27 times greater than that of non-transgenic plants, respectively. In contrast, the dry weight of RNA interference-expressing plants (Ri-1 and Ri-4) was reduced by 21% and 19%, respectively, when compared to the non-transgenic potato lines. Correspondingly, 7 d after heat treatment, the root dry weight of overexpressing *StATG18a* plants (OE-2 and OE-5) exhibited an increase of 43% and 52%, respectively, relative to non-transgenic plants. The root dry weight of RNA interference-expressing plants (Ri-1 and Ri-4) was found to be decreased by 6% and 2% than those of non-transgenic potato plants, respectively. Similarly, 14 d after heat treatment, the root dry weight of overexpressing *StATG18a* plants (OE-2 and OE-5) was determined to be increased by 1.33-fold and 1.28-fold than those of non-transgenic plants, respectively. When compared to non-transgenic plants, the root dry weight of RNA interference-expressing plants (Ri-1 and Ri-4) was reduced by 20% and 16%, respectively. In summary, the *StATG18a* has been shown to mitigate the detrimental effects of heat stress and plays a crucial regulatory role in the growth and development of potatoes under such stress conditions.

### 3.9 *StATG18a* affected the physiological indexes of potato plants under heat stress conditions

**Figure 5:**
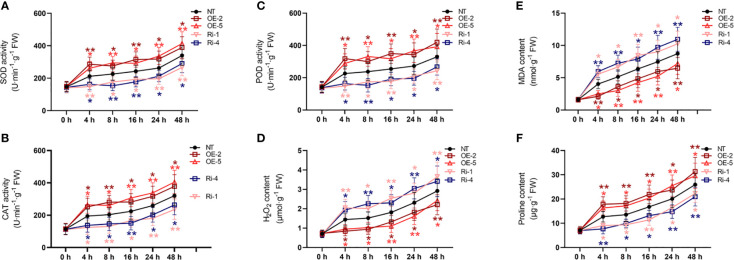
Effects of *StATG18a* on **(A)** SOD activity, **(B)** CAT activity, **(C)** POD activity, **(D)** H_2_O_2_ content, **(E)** MDA content, and **(F)** proline content. Potato plants (transgenic and non-transgenic) were treated with heat stress at different time intervals (0, 4, 8, 16, 24, and 48h), and examined the SOD, CAT, as well as POD activities, and H_2_O_2_ MDA as well as proline content. NT, non-transgenic plants; NT, non-transgenic plants; OE, pBI121-EGFP-StATG18a-transgenic plants (OE-2 and OE-5); Ri, pART-StATG18a-RNAi-transgenic plants (Ri-1 and Ri-4); Data were computed by means ± standard deviation; p-values (*p<0.05, **p<0.01) were calculated by ordinary two-way ANOVA, followed by Tukey’s multiple comparisons tests (n = 9).

Under normal conditions, compared with non-transgenic plants, the activities of SOD, CAT, and POD activities, as well as the amounts of H_2_O_2_, proline, and MDA, were not significantly changed in overexpressing *StATG18a* and knockdown potato plants as shown in **Figure 5**. Under heat stress conditions, the overexpression of *StATG18a* resulted in elevated levels of SOD activity (**Figure 5A**), CAT activity (**Figure 5B**), POD activity (**Figure 5C**), and proline content (**Figure 5D**). In contrast, the knockdown expression of *StATG18a* led to a reduction in the levels of SOD activity (**Figure 5A**), CAT activity (**Figure 5B**), POD activity (**Figure 5C**), and proline content (**Figure 5D**) as compared to the non-transgenic plants (*p < 0.05, **p < 0.01). Concurrently, we noticed a substantial decrease (*p < 0.05, **p < 0.01) in the contents of H_2_O_2_ (**Figure 5E**) and MDA (**Figure 5F**) in overexpression of *StATG18a* potato plants when subjected to heat stress, as compared to the non-transgenic plants. Meanwhile, we observed that the levels of H_2_O_2_ (**Figure 5E**) and MDA (**Figure 5F**) in potato plants overexpressing *StATG18a* were significantly reduced (*p < 0.05, **p < 0.01) during heat stress, as compared to the non-transgenic plants. However, we noticed the reverse results for the contents of H_2_O_2_ (**Figure 5E**) and MDA (**Figure 5F**) in the knockdown expression of *StATG18a* potato plants (*p < 0.05, **p < 0.01) (**Figures 5E, F**). The research showed that when exposed to heat stress, transgenic lines with overexpressing *StATG18a* had less physiological harm compared to the non-transgenic potato lines. Conversely, lines with RNA interference expression had more physiological damage than the NT lines. Therefore, *StATG18a* influenced the physiological functions of potato plants in response to heat stress.

### 3.10 *StATG18a* affected the photosynthesis of potato plants under heat stress conditions

**Figure 6:**
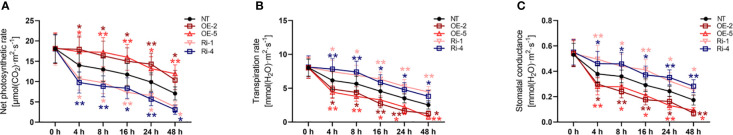
Effects of *StATG18a* on **(A)** net photosynthetic rate, **(B)** transpiration rate and **(C)** stomatal conductance under heat conditions.Potato tubers stimulated by transgenic and non-transgenic lines were monitored by cultivation with heat stress treatment at different time intervals (0h, 4h, 8h, 12h, 24h, and 48h). Analyses for heat stress **(A)** net photosynthetic rate, **(B)** transpiration rate, and **(C)** stomatal conductance. NT, non-transgenic plants; OE, pBI121-EGFP-StATG18a-transgenic plants (OE-2 and OE-5); Ri, pART-StATG18a-RNAi-transgenic plants (Ri-1 and Ri-4); Data were computed by means ± standard deviation; p-values (*p<0.05, **p<0.01) were estimated by ordinary two-way ANOVA, followed by Tukey’s multiple comparisons tests (n = 9).

Moreover, we observed that net photosynthetic rate (**Figure 6A**), transpiration rate (**Figure 6B**), and stomatal conductance (**Figure 6C**) were not significantly changed by *StATG18a* overexpression and knockdown compared to non-transgenic plants under normal conditions (*p > 0.05*). However, we detected that compared with non-transgenic plants net photosynthetic rate (**Figure 6A**) of potato plants with high overexpression of *StATG18a* increased, and the transpiration rate (**Figure 6B**) and stomatal conductance (**Figure 6C**) decreased under heat stress. Meanwhile, *StATG18a* knockdown decreased net photosynthetic rate (**Figure 6A**), while transpiration rate (**Figure 6B**) and stomatal conductance were increased (**Figure 6C**) (*p < 0.05, **p < 0.01) under heat stress conditions. On 48h of treatment, the net photosynthetic rates of OE-2 and OE-5 were 1.47 and 1.68 times more than those of NT plants, respectively, while Ri-1 and Ri-4 were increased by 53% and 43% compared to NT plants, respectively. On the contrary, the transpiration rate and stomatal conductance for all plants were decreased, the rate of that decline was faster in OE-2 and OE-5, and values were, given as 30%–49% and 40%–53%, respectively, than those of NT plants, while Ri-1 and Ri-4 were increased by 1.5–1.76 and 1.46–1.62 times, respectively, compared to NT plants. It is evident from the above statement that the *StATG18a* plays a significant function in photosynthesis by regulating the net photosynthetic rate, transpiration rate, and stomatal conductance in potato plants under heat stress.

## 4 Discussion

The prospect of exceedingly hot weather is increasing due to global climate change, and its length is steadily getting longer. This has detrimental effects on crop growth and development, especially in China’s north and northwest regions where temperatures can approach 35°C during the potato growing season. Heat Stress modifies the cellular processes, metabolism, physiology, photosynthesis, and phenotype of crop plants, which negatively impacts all stages of crop growth, development, yield, and productivity (Hasanuzzaman et al., 2013). The heat tolerance of cultivars may be assessed using these characteristics (Hastilestari et al., 2018). To overcome these problems, plants acquire several kinds of mechanisms and responses, one such mechanism is autophagy, which is regulated by several *ATGs* that have been discovered in many crop plants (Cao et al., 2021).

In this study, six putative genes belonging to the *StATG18* subfamily were identified by wide-genome analysis data of potatoes (**Supplementary Table 1**). In a previous study, a polygenetic analysis found 108 putative *TaATGs* from 13 different subfamilies in wheat. The subfamily *TaATG16* has 29 genes categorized based on conserved motifs and exon-intron composition. Moreover, 114 cis-elements were discovered which play a significant role in response to stress, light, and hormonal association (Yue et al., 2018). In our study, chromosomal localization and gene duplication were carried out to find the position of the member of the subfamily *StATG18* genes by phylogenetic investigation. Similarly, conservative motifs distribution and composition as well as intron-exon length and number were also analyzed in potatoes with other crop species, such as *Arabidopsis*, rice, and tomato. Finally, 37 cis-regulatory elements with different functions have been identified and the phylogenetic relationship between potato and other crop species was drawn by collinear analysis.

The qRT-PCR research demonstrated that the *StATG18* subfamily genes exhibited varying levels of expression in the different tissues (tuber, flower, petiole, stem, stolon, leaf, and root) of potato plants (**Figure 1**). It is hypothesized that these genes play a role in the growth and development of different tissues and organs in potato plants. Another research on autophagy-related genes of *Medicago truncatula* demonstrated that *MtATGs* have a potential role in the growth and development of plants. The expression profile of these genes was identified in various tested tissues and organs at various stages of seed development. Through microarray analysis, it is reported that the *MtATG* genes showed varied expression in roots, leaves, stems, and flowers in the late seed development stage (Benedito et al., 2008). Additionally, under heat stress, *StATG18* subfamily gene expression was also observed in roots, stems, and leaves of potato plants in our investigation (**Figure 2**). Furthermore, qRT-PCR analysis suggests that the *StATG18a* exhibited a high expression pattern in roots, stems, and leaves, after subjected to heat treatment, which was further analyzed for heat stress studies. Previous reports indicated that the results of qRT-PCR and RT-PCR showed that *TdATG8* is constitutively expressed in roots and leaves and it is increased in both tissues in response to osmotic stress and PEG-treated leaves and roots, respectively. It also exhibits 24–40 fold increases in *TdATG8* expression levels in roots and leaves under abiotic stress conditions (Kuzuoglu-Ozturk et al., 2012).

In a study, it was found that the subcellular localization investigation of GFP-SiATG8a determined the confirmation of *SiATG8a* protein in the cytoplasm as well as in the membrane in foxtail millet (*Setaria italica* L), while the GFP vector is used as a control (Li et al., 2015). In another investigation, the green fluorescence observation depicted the presence of MdHARBI1–GFP union protein in the nucleus of apples (*Malus domestica*), while GFP is used as a control vector, which was present in the nucleus, cytoplasm, and membrane (Huo et al., 2021). In our study, subcellular localization analysis validated the presence of StATG18a in the cytoplasm and nucleus. Through confocal scanning laser microscopy, the green fluorescence detected the protein of StATG18a in the cytoplasm and nucleus shown in **Figure 3**. The strong cytoplasmic and nuclear membrane signals from GFP-StATG18a further validated the presence of StATG18a in the cytoplasm and nucleus, whereas the empty or control vector the GFP was presented in the membrane, cytoplasm and the nucleus.

According to previous research reports, wild-type (WT) plants of apples were exposed to 48°C for 6 hours resulting in burned and wrinkled leaves. However, only the young leaves on top of transgenic plants showed indications of dehydration and burn, while mature leaves remained green and robust, and was found that heat stress prompted minor damage to *MdATG18a* overexpressing apple plants (Sun et al., 2018). High temperatures also caused leaf wilting and decreased leaf relative water content (RWC). Heat treatment reduced total chlorophyll concentrations, although the loss was less in transgenic plants compared to WT plants. Heat stress reduced the damage of phenotypes of transgenic apples, indicating that *MdATG18a* plays a vital role in heat stress response (Sun et al., 2018). Our phenotypic analysis of transgenic and non-transgenic potato plants showed that potato plants showed no significant differences in response to heat stress treatment and control conditions (**Figures 4C1, C2**). Compared with non-transgenic potato lines, the growth and progression of agronomic traits (such as total fresh and dry weight, root fresh and dry weight) of overexpressing *StATG18a* lines (including OE-2 and OE-5) were greatly improved 7 and 14 d after heat treatment. Most NT and knockdown potato plants were slow to recover from the effects of high temperature or their leaves turned yellow and wilted due to high-temperature burns (**Figures 4C3, C4**), but overexpressing *StATG18a* plants showed tolerance to high temperature and recovered relatively well, with few leaves wilting, which demonstrated the role of autophagy-related genes in potato plants under high-temperature stress.

Environmental stressors cause physiological changes in crop plants, and these changes are considerable enough to gauge a plant’s tolerance and resilience to different biotic and abiotic stress scenarios (Tang and Bassham, 2022). Autophagy-related genes are essential for maintaining genome integrity in eukaryotic species. This idea is further supported by the existence of up-regulated genes linked to DNA repair and responses to stimuli that cause DNA damage (Feng and Klionsky, 2017). In a prior investigation, the role of ATGs concerning physiological markers including MDA, H_2_O_2_, and proline content, as well as POD, SOD, and CAT activity, was evaluated under various abiotic stress conditions. Under salt and drought stress conditions, a significant difference was seen in the levels of proline, MDA, and H_2_O_2_ as well as in antioxidant enzyme systems (Zhou et al., 2015). Physiological characteristics were examined in this work to evaluate the *StATG18a* gene’s tolerance to heat stress. Because heat stress causes an increase in H_2_O_2_ and MDA concentration and alters the equilibrium of reactive oxygen species (ROS), the *StATG18a*-overexpression potato lines displayed a downward trend in these cases. Therefore, overexpression of *StATG18a* in OE-2 and OE-5 potato plants under heat stress resulted in an improved MDA (**Figure 5E**) and H_2_O_2_ (**Figure 5D**) content, whereas RNA interference expression lines (Ri-1 and Ri-4) displayed elevation in comparison to non-transgenic lines. Overall, the regulating role of overexpressing *StATG18a* potato plants under heat stress is confirmed by the considerable variation in physiological parameters.

Heat stress induces morphological and structural alterations in leaves, the primary organ engaged in transpiration and photosynthesis, resulting in wilting and changes to morphology, anatomy, physiology, and photosynthetic ability (Mathur et al., 2014). Plant photosynthesis is significantly influenced by several factors such as the rate of transpiration and the absorption of CO_2_ by leaves, which are both regulated by stomatal behavior (Hu et al., 2018). In a previous report, after four hours of heat treatment, differences in stomatal morphology between overexpressing *MdATG18a* and WT plants were observed in apples. The stomatal aperture was reduced by a high temperature, although the OE lines had less shrinking overall (Huo et al., 2020). It also examined the gaseous exchange characteristics of the overexpressing *MdATG18a* plants following an 18-hour recovery from heat treatment to assess the extent of damage in this respect. No variation in Pn, Gs, Ci, or Tr was seen across plants of various genotypes under normal temperature conditions. Pn was significantly reduced in plants of all genotypes; however, the two overexpressing *MdATG18a* lines’ Pn differences were approximately 1.74 times larger because of the overexpression of the *MdATG18a* gene than those of the WT plants (Huo et al., 2020). In our study similar results were observed, maximum net photosynthesis, transpiration, and stomatal conductance was exposed in all potato lines (transgenic and non-transgenic) under normal conditions. After 4–48 h of heat treatment, the OE-2 and OE-5 transgenic potato lines indicated an upward trend in net photosynthetic rate (**Figure 6A**) compared to non-transgenic lines due to overexpression of *StATG18a* gene, while knockdown expression of *StATG18a* gene revealed contrary results. Transpiration rate (**Figure 6B**) and stomatal conductance (**Figure 6C**) were inhibited due to the overexpression of the *StATG18a* gene after heat stress treatment compared to non-transfected lines. It is very crucial to maintain water balance in plants under high temperatures and dry conditions to avoid wilting. Stomatal behavior directly regulates the rate of transpiration under heat or drought stress, it is very essential to preserve water loss under high temperatures to uphold water equilibrium in plants. Therefore, overexpression of *StATG18a* showed a stronger downward trend in transpiration rate and stomatal conductance to preserve water under heat stress, whereas knockdown expression induces heat stress by exceeding the transpiration rate by increasing stomatal conductance. These confirmations propose that the photosynthetic indices of overexpressing *StATG18a* potato lines OE-2, and OE-5 depicted enhanced progression under heat stress treatment, compared to non-transgenic lines.

## 5 Conclusion

In conclusion, we identified the *StATG18* subfamily genes in the potato genome. Firstly, a total of 6 putative subfamily *StATG18* members were systematically analyzed for the phylogenetic relationships, chromosome distribution and gene replication, conserved motifs, gene structure, interspecific collinearity relationship, and cis-regulatory elements. Secondly, the expression profile of *StATG18* subfamily genes by qRT-PCR demonstrated the differential expression level in different potato parts (leaf, flower, stem, root, stolon, petiole, and tuber). Additionally, the expression pattern of roots, stems, and leaves was also investigated under heat stress, which validated the high expression level of the *StATG18a* gene in leaves, stems, and roots of potato plants under heat treatment at different time intervals. Finally, our results showed the localization of StATG18a in the cytoplasm and nucleus by green florescence from EGFP expressed by pBI121-EGFP-StATG18a through subcellular localization analysis. Moreover, physiological and photosynthetic indices also depicted the highly significant differences in overexpressing *StATG18a* lines (OE-1 and OE-5), compared to non-transgenic potato lines under heat stress. Additionally, *StATG18a* modulated the growth of transgenic plants, as depicted after the 7^th^ and 14^th^ d of heat treatment the OE-1 and OE-5 potato plants showed less injury and wilting than those of non-transgenic plants. This study not only provides novel insight into *StATG18* subfamily members’ characterization and functional identification of potato autophagy but also provides essential evidence about the *StATG18*a gene functional analysis under heat stress in potato plants.

## Data Availability

The datasets presented in this study can be found in online repositories. The names of the repository/repositories and accession number(s) can be found in the article/**Supplementary Material**.
