# Supplementary material for: Identification of autophagy-related genes ATG18 subfamily genes in potato (Solanum tuberosum L.) and the role of StATG18a gene in heat stress
Source: Front Plant Sci. 2024 Aug 27;15:1439972. doi: 10.3389/fpls.2024.1439972 (PMC11387889; doi:10.3389/fpls.2024.1439972)
Supplement: Supplementary file 1 [file DataSheet1.docx]

1. **List of supplementary Tables**

**Accession Numbers for potato, *Arabidopsis*, rice, and tomato.**

The ATG sequences discussed in this article can be accessed at The *Arabidopsis* Information Resource (<https://www.arabidopsis.org/>), The Rice Genome Annotation Project (http://rice.uga.edu /index.shtml) and Phytozome (<https://phytozome-next.jgi.doe.gov/info/Slycopersicum_ITAG4_0>) under the specified accession number:

***Arabidopsis***

AtATG18a (At3g62770), AtATG18b (At4g30510), AtATG18c (At2g40810), AtATG18d (At3g56440), AtATG18e (At5g05150), AtATG18f (At5g54730), AtATG18g (At1g03380), AtATG18h (At1g54710),

**Rice**

OsATG18a (LOC_Os02g54910), OsATG18b (LOC_Os01g07400), OsATG18c (LOC_Os01g70780), OsATG18d (LOC_Os05g07710), OsATG18e (LOC_Os01g57720), OsATG18f (LOC_Os05g33610),

**Tomato**

SlATG18a (Solyc08g006010), SlATG18b (Solyc07g006120), SlATG18c (Solyc01g099400),

SlATG18f (Solyc12g005230), SlATG18g (Solyc01g098430), SlATG18h (Solyc07g064060).

**Table S1.** Features of identified *StATG18* subfamily genes in potato (*Solanum tuberosum* L.)

| **Gene name** | **Protein ID** | **Gene ID** | **Chr.** | **Start** | **End** | **Strand** | **AAL (aa)** | **MW (Da)** | **pI** | **(GRAVY)** | **Subcellular Localization** |
| --- | --- | --- | --- | --- | --- | --- | --- | --- | --- | --- | --- |
| StATG18a | PGSC0003DMP400026133 | PGSC0003DMT400038462 | Ch08 | 2664563 | 2669672 | - | 419 | 46750.24 | 8.44 | -0.217 | Cytoplasm, Nucleus |
| StATG18b | PGSC0003DMP400019718 | PGSC0003DMT400028996 | Ch07 | 1493132 | 1499092 | + | 365 | 39707.58 | 7.67 | 0.131 | Nucleus |
| StATG18c | PGSC0003DMP400042753 | PGSC0003DMT400063510 | Ch01 | 7.7E+07 | 7.7E+07 | + | 444 | 48536.61 | 6.22 | -0.213 | Nucleus |
| StATG18d | PGSC0003DMP400051539 | PGSC0003DMT400076107 | Ch08 | 4.5E+07 | 4.5E+07 | - | 428 | 47856.03 | 6.67 | -0.259 | Nucleus |
| StATG18f | PGSC0003DMP400044395 | PGSC0003DMT400065820 | Ch12 | 8301860 | 8306414 | - | 869 | 94198.61 | 6.83 | -0.283 | Chloroplast, Nucleus |
| StATG18h | PGSC0003DMP400033405 | PGSC0003DMT400049486 | Ch07 | 5.4E+07 | 5.4E+07 | - | 878 | 95453.87 | 6.3 | -0.281 | Chloroplast |

**Table S2.** Ka/Ks analysis and estimate of the absolute dates for the duplication events between the duplicated StATG18 subfamily genes in potato

| **Duplicated pair** | **Duplicate type** | **Ka** | **Ks** | **Ka/Ks** | **Divergence time (million years)** |
| --- | --- | --- | --- | --- | --- |
| StATG18a/StATG18d | Segmental | 0.220079 | 0.739225 | 0.297716 | 24.64081994 |

**Table S3.** The conservation motifs in *StATG18* subfamily genes in potato.

| **The conservation motifs in ATG18 subfamily genes** | |
| --- | --- |
| **motif1** | IKQIRAHDSPIAALALDPSGTLLATASTKGTLIRIFNIMDG |
| **motif2** | HLQEVRRGLDRAEIYSIAFSPNSQWLAVSSDKGTVHVFSLK |
| **motif3** | GGIGIVEMLFRSNILALVGGGDNPQYPPNKVMIWDDHQ |
| **motif4** | HDQVLWAGFDKLECEGGSTRQVLLLGYQYGFQVWDVEDADNVRELVSRHD |
| **motif5** | GVLPKYFSSEWSVAQFRLPEGTQYIVAFGH |
| **motif6** | RFYSLRSHSYVHQLRFRSTVYLVRCSSRVVAVLQAAQIHCFDAATLENEY |
| **motif7** | NDLKLLHQIETVANPKGLCAVSQHSGSSVLACPGLQKGQ |
| **motif8** | KNTVAILGLDGSFYRCKFDPVNGGEMTQLEYHNFLKSEE |
| **motif9** | SFPSSAPNGSLVAHYAKESSKQLAAGIINLGDMGYKKLSKYCSDLLPDGN |
| **motif10** | FNQDYSCFAAGTDHGFRIYNCDPFRETFR |

**Table S4.** Number statistics of different 37 cis-regulatory elements in promoter regions of *StATG18* subfamily genes.

| **Gene name** | **3** | **4** | **5** | **6** | **7** | **8** | **9** | **10** | **11** | **12** | **13** | **14** | **15** | **17** | **18** | **20** | **22** | **23** | **25** | **26** | **28** | **29** | **31** | **32** | **33** | **34** | **37** | **1, 2, 16, 19, 21, 24, 27, 30, 34, 35** |
| --- | --- | --- | --- | --- | --- | --- | --- | --- | --- | --- | --- | --- | --- | --- | --- | --- | --- | --- | --- | --- | --- | --- | --- | --- | --- | --- | --- | --- |
| *StATG18a* | 0 | 4 | 2 | 3 | 16 | 0 | 60 | 0 | 0 | 2 | 0 | 7 | 3 | 0 | 2 | 0 | 0 | 0 | 1 | 0 | 0 | 1 | 0 | 0 | 1 | 0 | 0 | 0 |
| *StATG18b* | 0 | 1 | 0 | 1 | 20 | 0 | 43 | 0 | 0 | 0 | 0 | 2 | 4 | 1 | 6 | 1 | 1 | 1 | 0 | 0 | 0 | 0 | 0 | 0 | 0 | 0 | 0 | 0 |
| *StATG18c* | 0 | 0 | 2 | 0 | 19 | 1 | 38 | 0 | 1 | 1 | 0 | 1 | 1 | 0 | 0 | 0 | 2 | 1 | 1 | 0 | 0 | 1 | 0 | 0 | 0 | 0 | 1 | 0 |
| *StATG18d* | 1 | 3 | 1 | 5 | 14 | 1 | 47 | 0 | 0 | 1 | 1 | 0 | 7 | 0 | 0 | 0 | 0 | 1 | 0 | 0 | 1 | 0 | 1 | 0 | 0 | 0 | 0 | 0 |
| *StATG18f* | 2 | 0 | 2 | 0 | 21 | 0 | 32 | 1 | 0 | 2 | 1 | 4 | 0 | 0 | 0 | 0 | 0 | 0 | 0 | 0 | 0 | 0 | 0 | 0 | 0 | 1 | 0 | 0 |
| *StATG18h* | 1 | 1 | 1 | 1 | 16 | 0 | 50 | 0 | 0 | 2 | 0 | 2 | 1 | 0 | 0 | 0 | 2 | 0 | 0 | 4 | 0 | 0 | 0 | 1 | 0 | 0 | 1 | 0 |

1=auxin-responsive element, 2= cis-acting element involved in low-temperature responsiveness, 3=cis-acting element involved in salicylic acid responsiveness, 4= cis-acting element involved in the abscisic acid responsiveness, 5= cis-acting regulatory element essential for the anaerobic induction, 6= cis-acting regulatory element involved in light responsiveness, 7= common cis-acting element in promoter and enhancer regions, 8= cis-acting regulatory element involved in zein metabolism regulation, 9= core promoter element around -30 of transcription start, 10= element involved in differentiation of the palisade mesophyll cells, 11= enhancer-like element involved in anoxic specific inducibility, 12=light responsive element, 13= MYBHv1 binding site, 14= part of a conserved DNA module involved in light responsiveness, 15= part of a light responsive element, 16= part of a light responsive module, 17= binding site of AT-rich DNA bind wing protein (ATBP-1), 18= cis-acting regulatory element involved in the MeJA-responsiveness, 19= cis-acting regulatory element related to meristem expression, 20= protein binding site, 21= MYB binding site involved in light responsiveness, 22= cis-acting element involved in defense and stress responsiveness, 23= cis-regulatory element involved in endosperm expression, 24= wound-responsive element, 25= gibberellin-responsive element, 26= MYB binding site involved in flavonoid biosynthetic genes regulation, 27= MYB binding site involved in flavonoid biosynthetic genes regulation, 28= part of a module for light response, 29= cis-acting regulatory element involved in circadian control, 30= cis-acting regulatory element involved in seed-specific regulation, 31= cis-acting element involved in cell cycle regulation, 32= cis-acting element involved in light responsiveness, 33= element for maximal elicitor-mediated activation (2copies), 34= cis-acting regulatory element, 35= cis-acting regulatory element involved in auxin responsiveness, 36= responsive element to auxin-free medium, 37= cis-acting element involved in gibberellin-responsiveness.

**Table S6.** Specific primers were constructed for PCR and qRT-PCR.

| **Gene name** | **NCBI Number** | **Forward primer** | **Reverse primer** |
| --- | --- | --- | --- |
| *StEf1α* | XM_006347752.2 | GGTTGTATCTCTTCCGATAAAGGC | GGTTGTATCTCTTCCGATAAAGGC |
| *StATG18a* | [XM_006356959.2](https://www.ncbi.nlm.nih.gov/nucleotide/971567984?report=gbwithparts) | TCATGAGGGGAATGTGCTGG | AGCACCTCTCCTTACCTCCT |
| *StATG18b* | [XM_006343524.2](https://www.ncbi.nlm.nih.gov/nucleotide/971543842?report=gbwithparts) | GGTGCCGAATCCAAAAGGACT | CAGTGCAAACGGAGGTCAGA |
| *StATG18c* | [XM_006344584.2](https://www.ncbi.nlm.nih.gov/nucleotide/971545738?report=gbwithparts) | AGGGTCAGGTGAGAGTGGAA | CGCCTCTTCTGACCTCTTGG |
| *StATG18d* | [XM_006355156.2](https://www.ncbi.nlm.nih.gov/nucleotide/971564274?report=gbwithparts) | GCTCTTACGAGGGAAGGCAA | AACCCCTTCTTACCTCCTGC |
| *StATG18f* | [XM_006363461.2](https://www.ncbi.nlm.nih.gov/nucleotide/971579407?report=gbwithparts) | TGAGCATGATCAGGTTCTGTG | AACAGGACCATCTGACCTGGA |
| *StATG18h* | [XM_006360034.2](https://www.ncbi.nlm.nih.gov/nucleotide/971573314?report=gbwithparts) | GGTCTGACTAACGCGGTCAT | ACGACGTGCATCAGATGTGT |
| **Overexpression:** | | | |
| *StATG18a* | [XM_006356959.2](https://www.ncbi.nlm.nih.gov/nucleotide/971567984?report=gbwithparts) | CTCGAGATGGCCACTGTTTCCCCTCTCC | GTCGACCGAGGCCTTTTCGGACTTCAGAA |
| **RNA Interference expression:** | | | |
| *StATG18a* | [XM_006356959.2](https://www.ncbi.nlm.nih.gov/nucleotide/971567984?report=gbwithparts) | TGGCCACTGTTTCCCCTCTCC | GTAAGGGTCACAACTATAGACA |

1. **List of supplementary figures.**

**
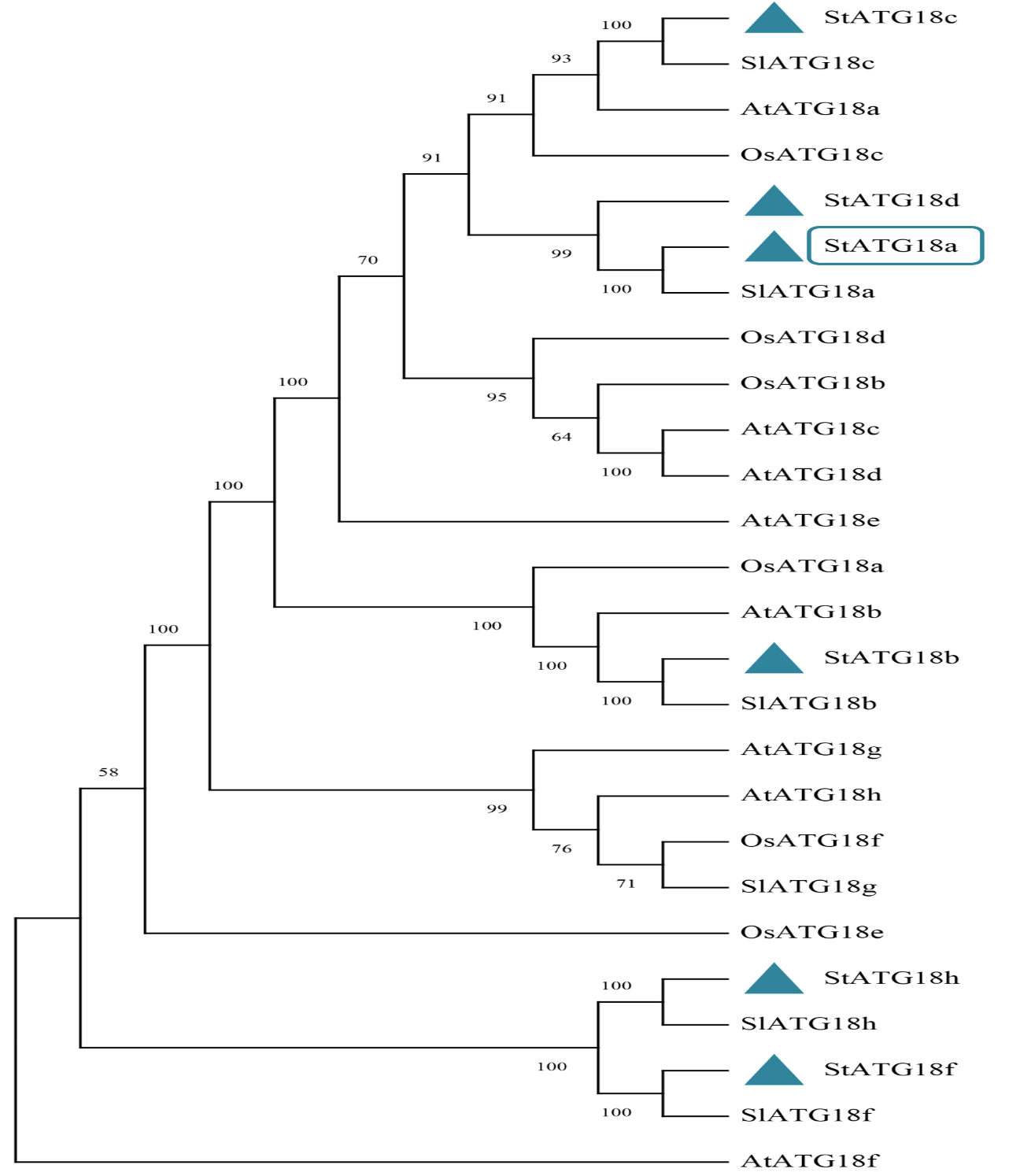
**

**Fig. S1.** Phylogenetic investigation of potato *StATG18* subfamily genes with other crop species. Phylogenetic interactions of StATG18 subfamily genes from *Solanum tuberosum* (St) with those of Arabidopsis *thaliana*, (At), Oryza sativa (Os), and *Solanum lycopersicum* (Sl). By utilizing the Neighbor-Joining (NJ) method with 1000 bootstraps, the tree was fabricated by MEGA 6.0 software. In each tree, potato StATG18 subfamily genes are indicated by the blue triangle.


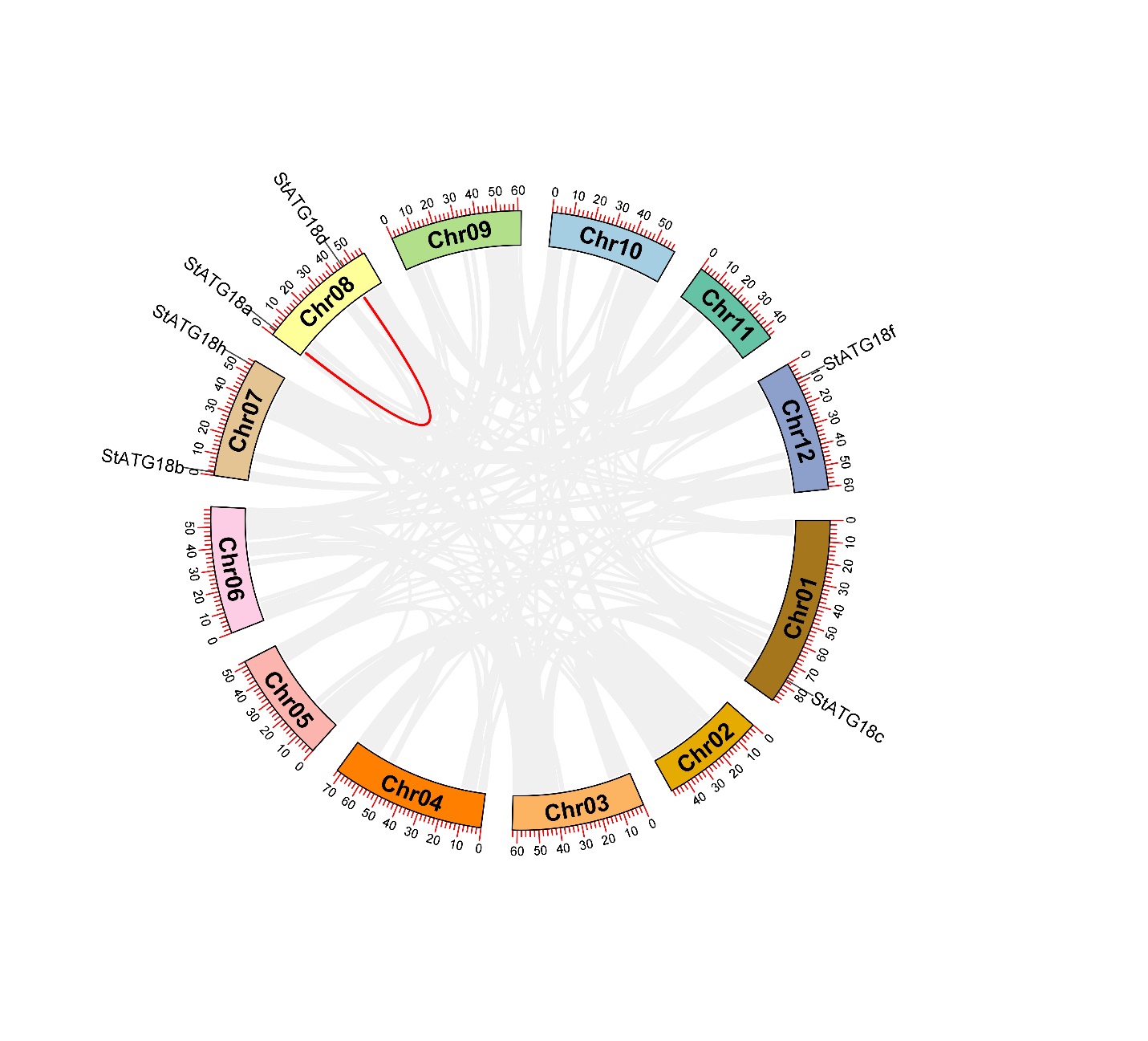


**Figure S2.** Chromosomal distribution and gene duplication of *StATG18* subfamily genes. Chromosomal distribution of *StATG18* subfamily genes was conducted by Circos software. Gene duplication events of *StATG18* subfamily gees were analyzed by MCScanX. The gray lines inside represent all synteny blocks in the potato genome. The segmentally duplicated genes are linked by the bold red line.


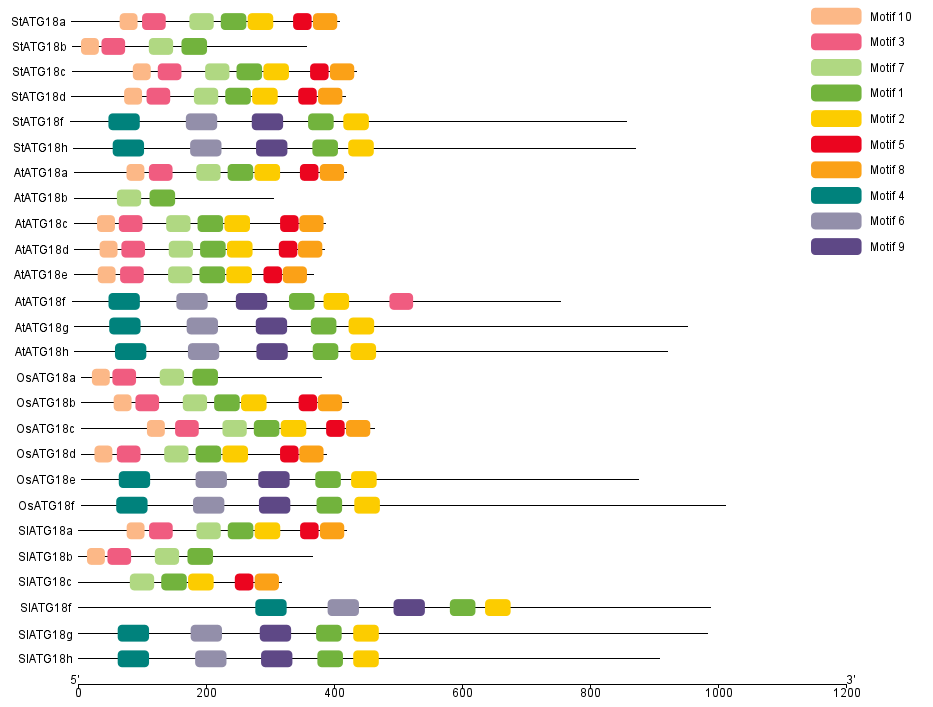


**Figure S3**. Conservative motifs analysis of *StATG18* subfamily genes of potato with other crop species (*Arabidopsis*, rice, and tomato). Each family contains different numbers of conserve motifs, the conserve motifs ranged from 1-10, which are depicted in different colors.


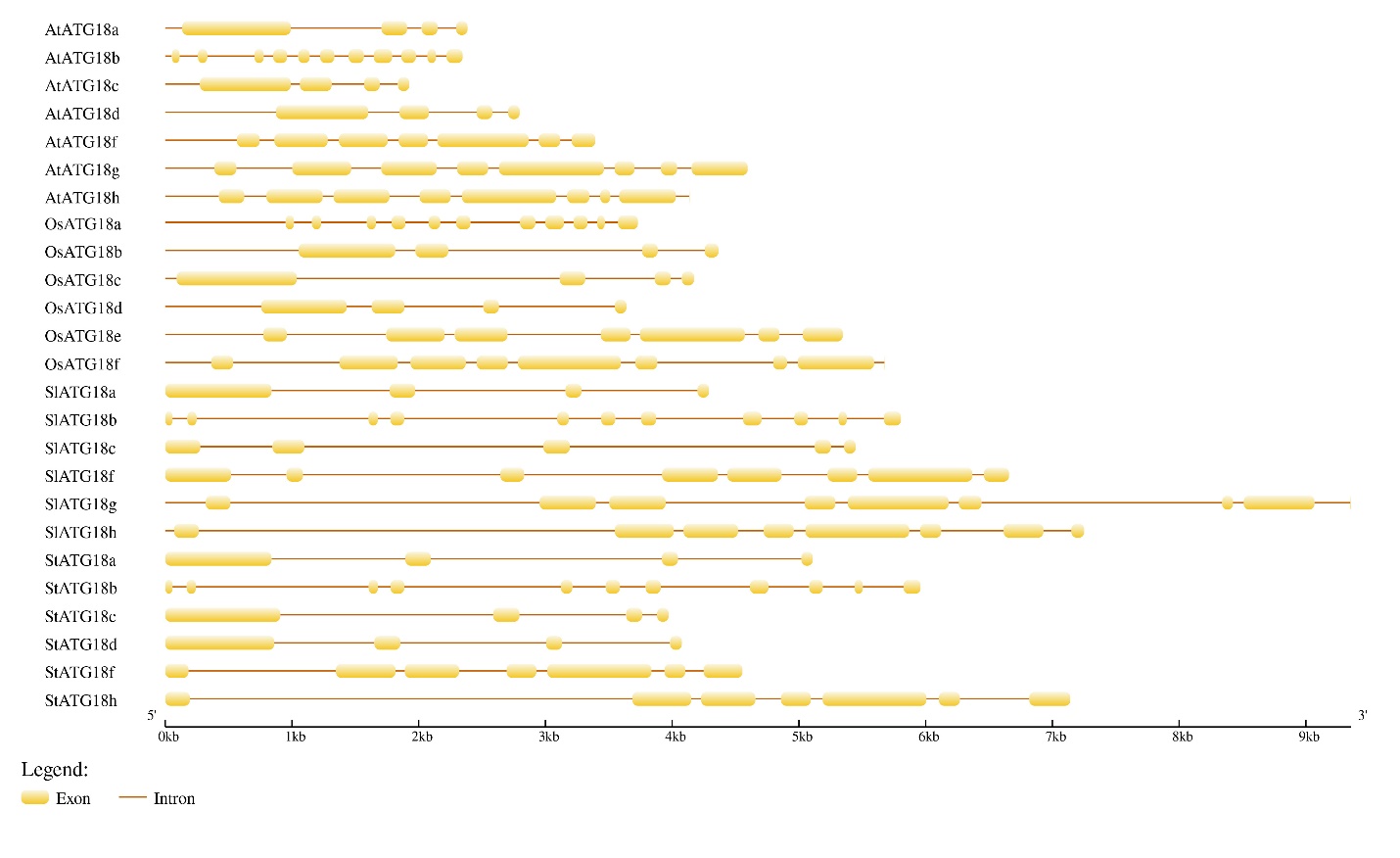


**Figure S4.** The exon-intron structure of the 6 StATG18 subfamily genes of potato, *Arabidopsis*, rice, and tomato. The exon-intron structures of the *StATG18* and other species were determined by comparing the coding sequences and the corresponding genomic sequences using the Gene Structure Display Server (GSDS) program. Yellow color bars are indicated exons and brown colored lines are intron, whereas exon numbers ranged from 4-11.

**
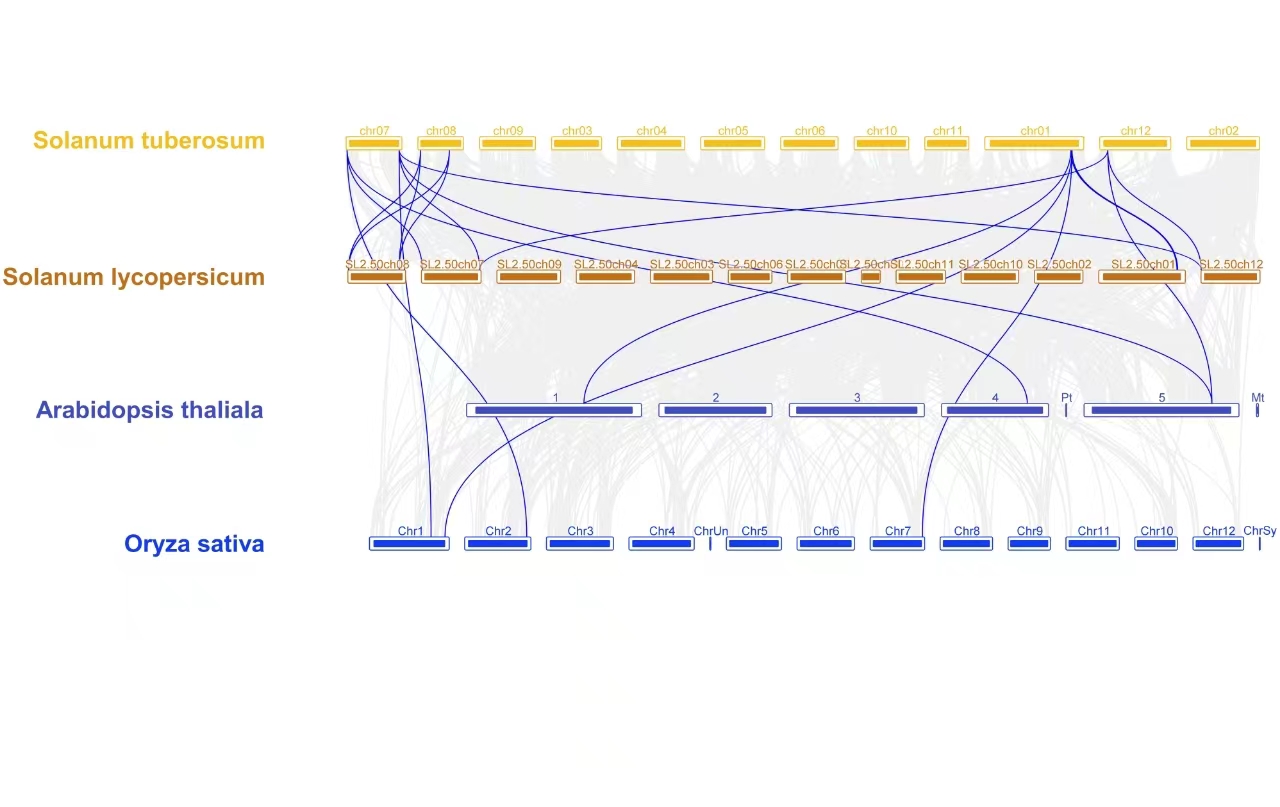
**

**Fig. S5.** Interspeciﬁc collinearity relationship between ATG18 subfamily members in potato, tomato, Arabidopsis, and rice. The chromosomes of *Solanum tuberosum*, *Solanum lycopersicum* , *Arabidopsis thaliana,* and *Oryza sativa* are marked with different colors. The collinear relationship of *StATG18* subfamily genes from *Solanum tuberosum* with those of *Arabidopsis thaliana,* *Oryza sativa*, and *Solanum lycopersicum*. The collinear relationship was analyzed by MCScanX software. Gray lines in the background indicate the collinear blocks within potato and other plant species' genomes, while the blue lines highlight the collinear *StATG18* subfamily gene pairs.

**
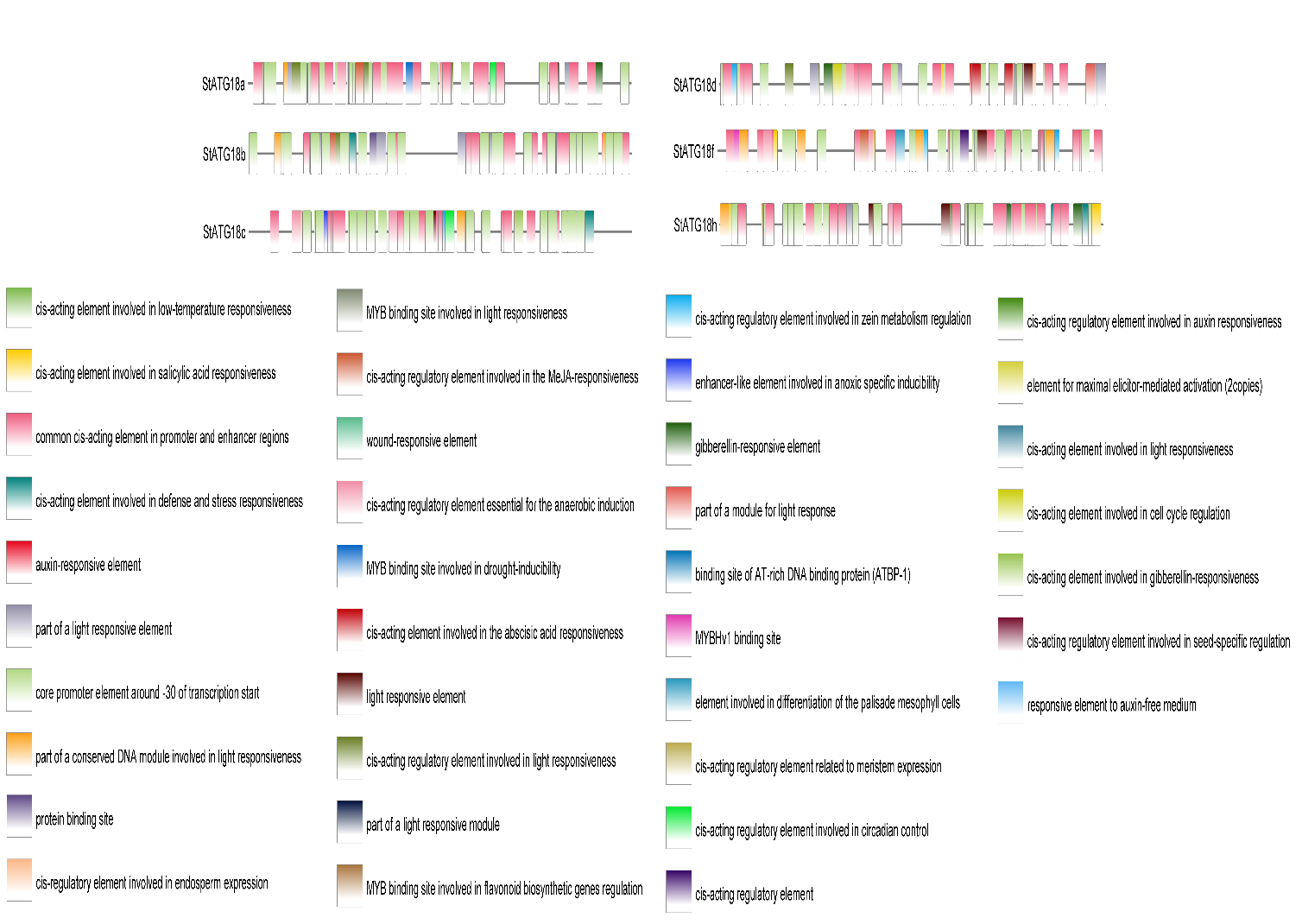
**

**Figure S6**. The schematic representation of cis-regulatory elements present in the promoter regions of *StATG*18 subfamily genes. 37 *cis*-elements were identified in 6 *StATG18* subfamily genes of potatoes with different structures and functions for each *StATG18* subfamily genes divided into 4 categories light responsiveness related, MYB-binding sites, hormone-related, and stress-related.
